# Kerfdr: a semi-parametric kernel-based approach to local false discovery rate estimation

**DOI:** 10.1186/1471-2105-10-84

**Published:** 2009-03-16

**Authors:** Mickael Guedj, Stephane Robin, Alain Celisse, Gregory Nuel

**Affiliations:** 1 Statistics and Genome laboratory, CNRS UMR8071, INRA U1152, University of Evry, Evry, France; 2 AgroParisTech, Statistics and Genome group, UMR INRA 518, Paris, France; 3 University Paris Descartes, MAP5, UMR CNRS 8145, Paris, France; 4 Statistics for Systems Biology Group, Paris, France

## Abstract

**Background:**

The use of current high-throughput genetic, genomic and post-genomic data leads to the simultaneous evaluation of a large number of statistical hypothesis and, at the same time, to the multiple-testing problem. As an alternative to the too conservative Family-Wise Error-Rate (FWER), the False Discovery Rate (FDR) has appeared for the last ten years as more appropriate to handle this problem. However one drawback of FDR is related to a given rejection region for the considered statistics, attributing the same value to those that are close to the boundary and those that are not. As a result, the local FDR has been recently proposed to quantify the specific probability for a given null hypothesis to be true.

**Results:**

In this context we present a semi-parametric approach based on kernel estimators which is applied to different high-throughput biological data such as patterns in DNA sequences, genes expression and genome-wide association studies.

**Conclusion:**

The proposed method has the practical advantages, over existing approaches, to consider complex heterogeneities in the alternative hypothesis, to take into account prior information (from an expert judgment or previous studies) by allowing a semi-supervised mode, and to deal with truncated distributions such as those obtained in Monte-Carlo simulations. This method has been implemented and is available through the R package kerfdr *via *the CRAN or at .

## Background

Multiple-testing problems occur in many bioinformatic studies where we considere a large set of biological objects (genes, SNPs, DNA patterns, etc.) and we want to test a null hypothesis H for each object. Typically, H may be 'the expression level of the gene is not affected by the treatment' or 'the pattern is as frequent as expected in the observed DNA sequence'. The control of the number of false positives, *i.e. *falsely rejected hypotheses, is the crucial issue in multiple testing. To this end, several error rates, such as the Family-Wise Error-Rate (FWER) or the False Discovery Rate (FDR), have emerged and various strategies to control these criteria have been developed (see [1] for a review).

In the last decade the FDR criterion introduced in [2] has received the greatest focus, due to its lower conservativeness compared to the FWER. The FDR is defined as the mean proportion of false positives among the list of rejected hypotheses. It is therefore a global criterion that cannot be used to assess the reliability of a specific hypothesis, *i.e. *that of a given gene, SNP or pattern.

More recently, a strong interest has been devoted to the local version of the FDR, called 'local FDR' [3] and denoted hereafter ℓ*FDR*. The idea is to quantify the probability for a given null hypothesis to be true. Even if many different strategies were designed to estimate the ℓ*FDR*, some of them based on the estimation of FDR itself [4], most of them rely on a mixture model assumption [5], which is a general and statistically convenient framework: the score (test statistics, *p*-values) on which the testing procedure is based follows a mixture distribution depending on the unobserved status of the hypothesis (true or false). Different approaches have been proposed: fully parametric [6-9], semi-parametric [10], Bayesian [11,12] or empirical Bayes [3].

The semi-parametric approach developed by [10] uses the knowledge of the distribution *f*_0 _of the score under the null hypothesis, to provide a flexible non-parametric estimation of the alternative distribution (denoted *f*_1_), *i.e. *under the alternative hypothesis. However, some important questions remain partially or not addressed in this reference.

In this paper we provide an implementation of the method with several important and practical generalizations. The Results and Discussion Section recalls the theoretical framework underlying our method, the properties of the estimation algorithm as well as the main steps of its implementation.

Performances are then studied via simulations, and compared to other existing methods. Finally, applications to various bioinformatic data sets, such as gene expressions, DNA sequence patterns and genome-wide associations, are carried out and proposed to the reader

## Results and discussion

### Semi-parametric mixture model

Our estimation of the local FDR (ℓ*FDR*) relies on the semi-parametric mixture model proposed in [10]. e have at our disposal *n *hypotheses {H_*i*_}_*i *= 1,...,*n *_we want to test. Suppose that an unknown proportion *π*_0 _of them are true nulls. For any hypothesis, we define a random variable *H*_*i *_that equals 0 if it is under **H**_0 _(true null hypothesis), and equals 1 under **H**_1 _(false null). For each H_*i*_, we compute a score denoted by *X*_*i *_(a *p*-value for example). We assume that these scores are independent and identically distributed, with mixture distribution

(1) *f*(*x*) = *π*_0 _*f*_0 _(*x*) + *π*_1 _*f*_1 _(*x*),

where *π*_1 _= 1 - *π*_0 _states for the proportion of false null hypotheses, *f*_0 _denotes the probability density function (pdf) of scores under **H**_0 _and *f*_1 _is the pdf of scores under **H**_1_. Note that *f*_0 _is completely specified. For instance if *X*_*i *_is the *p*-value of a Student statistic, *f*_0 _is the uniform distribution on [0, 1]. If any transformation (probit or log) is applied, *f*_0 _remains completely known. On the contrary, *f*_1 _needs systematically to be estimated so as to *π*_0_.

In our framework, ℓ*FDR *defined the probability that *H*_*i *_= 0 given the observed value *x*_*i *_of the score *X*_*i*_:

$$\ell FDR(x_{i})\overset{def}{\underset{}{=}}\tau_{i}=\mathrm{Pr}[H_{i}=0|X_{i}=x_{i}]=\frac{\pi_{0}f_{0}(x_{i})}{f(x_{i})}.$$

This quantity may be interpreted as a measurement of how likely the hypothesis at hand could be falsely rejected.

Since *f*_1 _is unknown, we use the following (non-parametric) kernel estimator for a given bandwidth *h *> 0

(2) $$\hat{f_{1}}(x)=\left[\sum_{i=1}^{n}\frac{H_{i}}{h}k\left(\frac{x-X_{i}}{h}\right)\right]/\left(\sum_{j=1}^{n}H_{j}\right),$$

in which we replace the unknown *H*_*i*_'s by their conditional expectation E [*H*_*i*_|*X*_*i*_] = Pr [*H*_*i *_= 1|*X*_*i*_] = 1 - *τ*_*i*_.

These expectations are themselves thanks to

(3) $$\hat{\tau_{i}}={\hat{\pi }}_{0}f_{0}(x_{i})/\hat{f}(x_{i}),$$

where ${\hat{\pi }}_{0}$ is a given estimator of the unknown proportion and $\hat{f}(x)={\hat{\pi }}_{0}f_{0}(x)+(1-{\hat{\pi }}_{0})\hat{f_{1}}(x)$. Thus, we obtain

(4) $$\hat{f_{1}}(x)=\left[\sum_{i=1}^{n}\frac{1-\hat{\tau_{i}}}{h}k\left(\frac{x-X_{i}}{h}\right)\right]/\left(n-\sum_{j=1}^{n}\hat{\tau_{j}}\right).$$

As $\hat{\tau_{i}}$'s and $\hat{f_{1}}$ depend on each other, we alternate the computation of (3) and (4) until convergence, which is proved in [10].

### Implementation

The method may require to apply a transformation to the sample of *p*-values (optional), to estimate the proportion of null hypotheses (*π*_0_), to determine an optimal value for the bandwidth (*h*) used in the kernel estimator and to compute the estimation of *f*_1_. These technical points are further developed and discussed in the Methods section.

Moreover, the corresponding R package allows a simple and straightforward use. For instance the command try = kerfdr(pv) for a given sample of *p*-values (pv) returns the estimates of *π*_0 _and ℓ*FDR *in try$pi0 and try$localfdr respectively. In addition the running time is very fast thanks to an efficient implementation using convolution through fast Fourier transforms and a list of customizable options for more advanced users such as the choice of *π*_0_, *h *or the kernel function. The complete R code and a pseudo-R code of kerfdr are available on the webpage.

### Practical generalizations

#### Semi-supervised cases

Prior information is actually available in many experiments. Among all the null hypotheses to be tested, some are known to be true (control genes in microarray experiments) while some others are known to be false (test genes in spike-in settings). Such a knowledge is taken into account in the estimation procedure described previously: known *a priori *the *τ*_*i*_s are kept fixed throughout the steps of the algorithm. They contribute to the estimation of *f*_1 _in Eq. (4), but are not updated in Eq. (3).

#### Truncation

Let us suppose now that we have at hand truncated data within an interval *I *= [*a*, *b*]. By 'truncated', we mean that the support of the *p*-values distribution is strictly smaller than [0,1]. For instance, if *B *denotes the number of simulations, *p*-values smaller than 1/*B *are often truncated to 0.0. How this will affect our method?

In order to deal with densities, the restrictions of *f*_0_, *f*_1 _and *f *to *I *need to be normalized. Denoting by *q*_0_, *q*_1 _and *q *the corresponding normalization factors, the mixture definition gives:

$$q=\int_{I}f(x)dx=\pi_{0}\underset{q_{0}}{\underset{︸}{\int_{I}f_{0}(x)dx}}+\pi_{1}\underset{q_{1}}{\underset{︸}{\int_{I}f_{1}(x)dx}}$$

Despite *q*_0_, *q*_1 _can not be easily computed as *f*_1 _is unknown. Fortunately, we can estimate *q *from a sample *X*_1_,..., *X*_*n *_of non-truncated data using

$$\hat{q}=\frac{1}{n}\sum_{i=1}^{n}I_{X_{i}\in I}$$

from which we derive

$$\hat{q_{1}}=\frac{\hat{q}-\pi_{0}q_{0}}{\pi_{1}}$$

One should note that this estimator does not necessarily belong to [0, 1]. In order to overcome this, we replace its value by 0 if $\hat{q_{1}}$ < 0 and by 1 if $\hat{q_{1}}$ > 1.

For example, if the *p*-values are estimated through Monte-Carlo using *B *= 500 simulations, the smallest non-null *p*-value is 1/*B *= 0.002 and *I *= [0.002, 1.000]. Let us assume that among a set of *n *= 1000 *p*-values, 54 are equal to 0.0, *π*_0 _= 0.9 and *π*_1 _= 0.1. We hence have $\hat{q}$ = (*n *- 54)/*n *= 946/1000 and as *q*_0 _= 1 - 1/*B *= 499/500 = 0.998 we easily get the expression of $\hat{q_{1}}$ (= 0.478).

### Simulation study

A comparison with other estimation methods of ℓ*FDR *is provided in [10]. It shows that the semi-parametric approach we propose performs as well as the empirical Bayes approach [13] and the Gaussian mixture model [8] when the distributions *f*_1 _and *f*_0 _are well separated. However, it outperforms them in more difficult situations, especially in terms of stability. We focus here on the particular cases described below (semi-supervised and truncation) that are not handle by the aforementioned methods.

#### Simulation design

We simulated sets of *p*-values according to the mixture model (1), where *f*_0 _is the uniform distribution over [0; 1]. We considered 4 different proportions of false null hypotheses (1 - *π*_0 _= 0.01, 0.05, 0.1 and 0.3), 2 different means for the *p*-values coming from the alternative distribution *f*_1 _(*μ *= 0.01 and 0.001). *f*_1 _is either an exponential distribution ℰ(1/*μ*) or a uniform distribution over [0, 2 *μ*]. The exponential distribution can provide values greater than one and a beta distribution as used in [6] can appear more appropriate; however it occurs very rarely with the taken value for *μ*. For each of the 4 × 2 × 2 = 16 configurations, *S *= 500 samples of size *n *= 1,000 were generated.

For each proportion *π*_0 _and distribution *f*_1_, the ℓ*FDR *of the *i*-th *p*-value *τ*_*i *_has a theoretical expression that is computed. Denoting by ${\hat{\tau }}_{i}^{s}$, the local FDR estimate of the *i*-th *p*-value for the simulation *s *(*s *= 1,..., *S*), the performances of the method are assessed by means of the root mean square error

$$RMSE(\pi_{0},f)=\frac{1}{S}\sum_{s}\sqrt{\frac{1}{n}\sum_{i}{\left({\hat{\tau }}_{i}^{s}-\tau_{i}\right)}^{2}}.$$

The smaller the *RMSE*, the better the performances.

#### Semi-supervised

**Figure 1:**
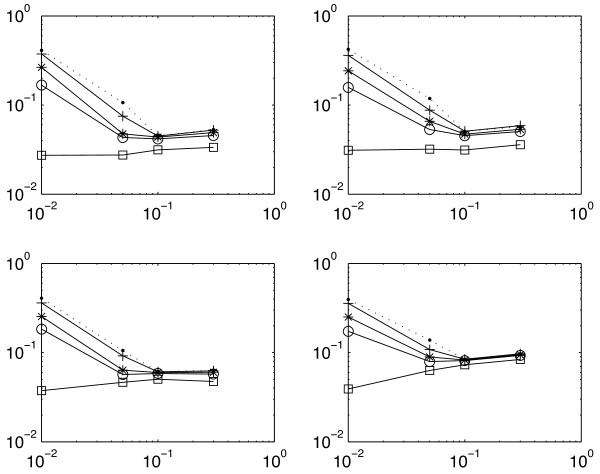
**Semi-supervised**. Root Mean Square Error (*RMSE*) between the true local FDR *τ *and the estimates as a function of the proportion 1 - *π*_0 _(log-log scale). Proportion of known hypothesis: *κ *= 0 (dotted), 1% (cross), 5% (asterix), 10% (circle) and 50% (square). Top: exponential shape for *f*_1_. Bottom: uniform shape. Left: *μ *= 0.001. Right: *μ *= 0.01. Variance of the *RMSE *lies between 1*e*^-4 ^and 5*e*^-4 ^with 500 simulations.

To see how prior information improves the estimation of ℓ*FDR*, we randomly select some hypotheses for which the status is known. The proportion *κ *of these hypotheses is fixed, so that the true value of the local FDR is also known (and equal either to 0 or 1). Figure 1 shows that even a small proportion (*κ *= 1% or 5%) of known hypotheses improves significantly the ℓ*FDR *estimation.

#### Truncation

**Figure 2:**
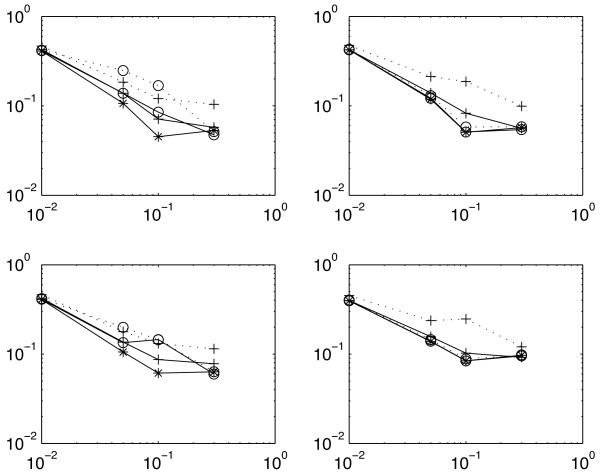
**Truncation**. Root Mean Square Error (*RMSE*) between the true local FDR *τ *and the estimates as a function of the proportion 1 - *π*_0 _(log-log scale). Truncation: p* = 0 (untruncated: asterix), 10^-3 ^(circle), 10^-2 ^(cross). Estimation: naive (dotted), corrected (solid). Top: exponential shape for *f*_1_. Bottom: uniform shape. Left: *μ *= 0.001. Right: *μ *= 0.01. Variance of the *RMSE *lies between 1*e*^-4 ^and 5*e*^-4 ^with 500 simulations.

In purpose of comparison, we truncate *p*-values to a given threshold p* (p* = 10^-2^, 10^-3^) and compare the generalized method that takes account of truncation with the naive one, in terms of the *RMSE *criterion. In Figure 2, the original non-truncated *p*-values provide a reference that can not be outperformed. We see that the correction improves the quality of the estimates, especially when the truncation is severe (p* = 10^-2^) and that the corrected estimates can be almost as good as the best achievable.

### Applications

#### Gene expression data

As a first illustration, we apply our method to the classical example of Hedenfalk [14] in which the expression levels of *n *= 3,226 genes are studied. The aim is to compare patients with two different breast cancers: 7 BRCA1 (7 patients) and BRCA2 (8 patients) corresponding to two different gene mutations predisposing to the disease. We use the modified *t*-test statistic proposed in [15] which avoids false-positives due to bad variance estimates.

**Figure 3:**
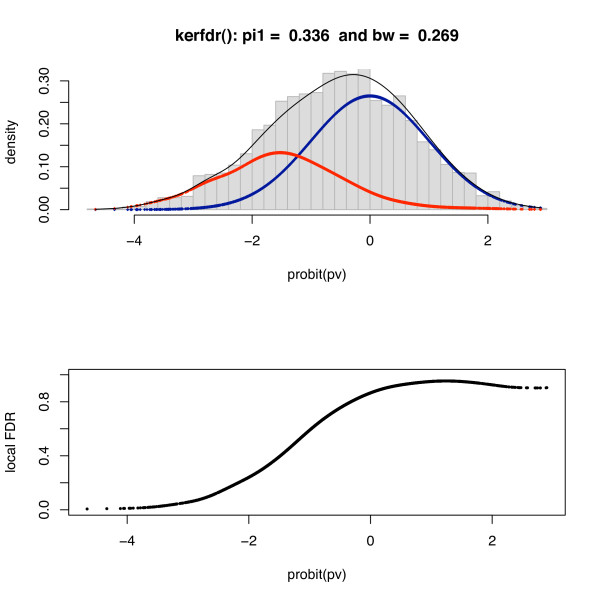
**Genes expression: estimated densities for the Hedenfalk dataset**. The expression levels of *n *= 3,226 genes for 7 BRCA1 and 8 BRCA2 patients (corresponding to two different gene mutations predisposing to the disease) are studied [14]; *p*-values are computed by using the modified *t*-test statistic proposed in [15].

Applying our method, we obtain a proportion of null genes of ${\hat{\pi }}_{0}$ = 66.4% which is consistent with the proportion estimated in [8] (${\hat{\pi }}_{0}$ = 65%). Figure 3 displays the estimated densities: although the proportion of modified genes is quite high (1 - ${\hat{\pi }}_{0}$ = 33.6%), the local FDR is lower than 1% for only 5 genes; it is below 5% for only 69. This shows that the local FDR is an efficient tool to reduce the type-I error-rate in difficult cases.

**Figure 4:**
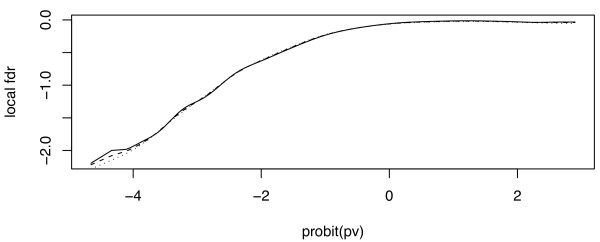
**Genes expression: sensitivity of local FDR estimates to the choice of the bandwidth**. *h *takes the values 0.20 (dotted), 0.27 (dashes) and 0.35 (line); local FDR are given in log_10 _scale.

The choice of the bandwidth is known to be a crucial step in density estimation problems. In this example, we selected a bandwidth of 0.27. To check to influence of this choice on the results, we tried several values of *h *between 0.20 and 0.35. Figure 4 shows that the estimated local FDR is not sensitive to this choice.

#### DNA sequence patterns

It is well known that most biological patterns in DNA sequences have unusual frequencies due to selection mechanisms. It is hence natural to search for new functional patterns among those whose number of occurrences is statistically significant. In order to do so, it is classical to adopt a test framework where the null hypothesis is that the DNA sequence is generated according to a order *m *⩾ 0 Markov model (the parameters of this Markov model are usually estimated over the observed sequence).

We consider here the complete genome of the pathogen bacteria *Mycoplasma genitallium *(575 kb) on which we estimate an order *m *= 3 homogeneous Markov model. For each of the 4^6 ^= 4,096 oligomers (DNA words) of length 6, we compute the exact expectation (E [*N*]) and standard deviation ($\sqrt{V[N]}$) of its frequency *N *from which we derive the z-score:

$$Z=\frac{N^{\text{obs}}-E[N]}{\sqrt{V[N]}}\overset{}{\underset{H_{0}}{\sim }}N(0,1)$$

where *N*^obs ^is the observed frequency of the oligomer in the genome.

Thanks to a simple CLT argument, we get that the distribution of *Z *is approximately a standard Gaussian under the null hypothesis. It is hence possible to use this approximation either by working directly with the z-score or by computing the two-sided *p*-value associated to each observation:

p-value=ℙ(N(0,1)<−|Z|)+ℙ(N(0,1)>+|Z|)

**Figure 5:**
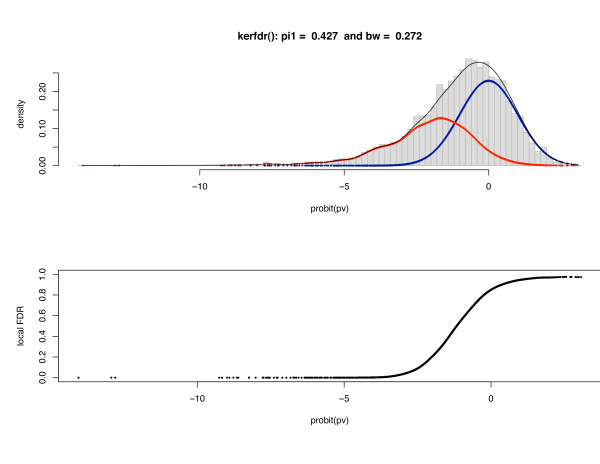
**Patterns in DNA sequences: estimated densities for all 4,096 oligomers of size 6 using *p*-values**. We consider here the complete genome of the pathogen bacteria *Mycoplasma genitallium *(575 kb); For each of the 4^6 ^= 4,096 oligomers of length 6, we compute the exact expectation (E [*N*]) and standard deviation ($\sqrt{V[N]}$) of its frequency *N *from which we derive the z-score and the corresponding *p*-value.

**Figure 6:**
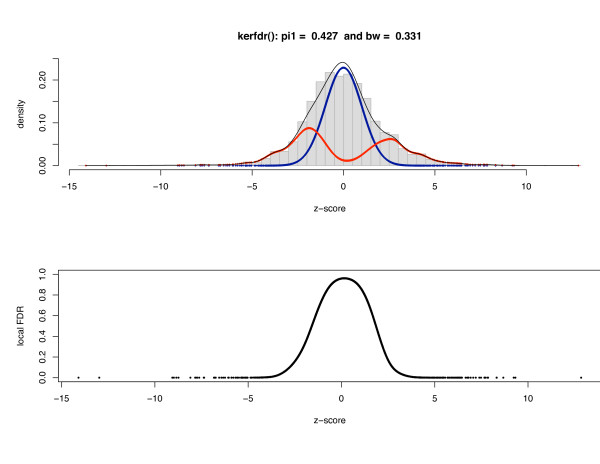
**Patterns in DNA sequences: estimated densities for all 4,096 oligomers of size 6 using z-scores**. This is the same dataset than Figure 5 with the difference that Local FDR is estimated from the z-scores directly instead of *p*-values. It results in a bimodal density for *f*_1_.

The natural approach is to estimate the densities from the *p*-values (Figure 5) where all the 'exceptional' oligomers (under and over-represented) accumulate on the left side of the resulting density. But the flexibility of our method allows us to make the estimations directly on the basis of the z-scores (Figure 6) by taking into account their bimodal distribution under **H**_1 _and distinguishing the oligomers that are under-represented (on the left side of the resulting density) from those that are over-represented (on the right side). If both strategies provide the same estimation for the proportion of 'null' oligomers (${\hat{\pi }}_{0}$ = 57.3%), ℓ*FDR *estimations are sensibly different in particular for the ligomers that are over-represented (data not shown).

#### Quality control in genome-wide association studies

In association studies, deviations from Hardy-Weinberg equilibrium (HWE) can be due to inbreeding, population stratification or selections. They can also be a symptom of lack of quality in genotyping because of a tendency to misscall heterozygous genotypes as homozygous for instance [16]. As a result, testing for HWE has often been proposed as a data quality check with the aim to discard loci that deviate from the equilibrium. Testing for deviations from HWE can be carried out using the Pearson chi-square statistic (*X*_HW_) that quantifies the distance between the observed genotype proportions and the ones expected under the equilibrium.

Here, the HWE test is applied to controls of genome-wide case-control data on the multiple sclerosis from France (Rennes). The data set consists in 74,067 Single Nucleotide Polymorphisms (SNPs). Since the usual chi-square approximation can be poor when there are low genotype counts, *p*-values are computed *via *Monte-Carlo simulations (number of simulations *B *= 10,000) which represents a typical case of truncation of *p*-values for those that are below the level of precision given by the number of simulations.

**Figure 7:**
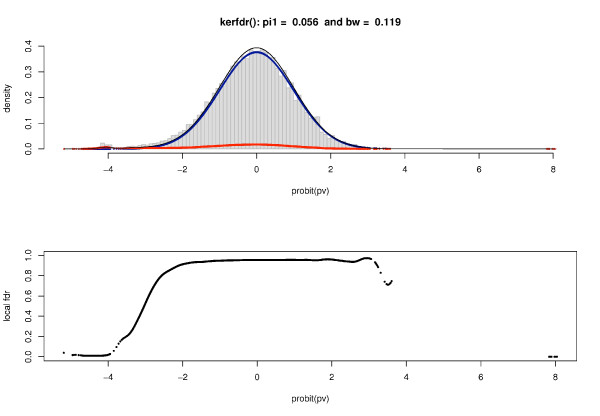
**Association studies: estimated densities for the Hardy-Weinberg test applied to a set of 74,067 SNPs**. DNA were genotyped using a 100 K Affymetrix chip. The algorithm used for making genotype calls has been previously described by Affymetrix. Local FDR is computed from the *p*-values resulting from an Hardy-Weinberg equilibrium test applied to each SNP. Note that *f*_0 _is almost perfectly overlapping *f *since *π*_0 _is close to 1.

Applying our method, we obtain a proportion of null SNPs of ${\hat{\pi }}_{0}$ = 99.44%. Figure 7 displays the estimated densities, showing a large overlap between the two distributions *f*_0 _and *f*_1_. By considering a threshold of 1%, then 29 SNPs would be declared to deviate from HWE, and up to 537 for a threshold of 5%. These quantities come down to 454 and 576 respectively when local FDR are estimated in the naive way (not accounting for the truncation). Consequently and in addition to our simulations, this application underlines an inflation of excluded SNPs when the information about a truncation, when it exists, is not taken into account in the estimation procedure.

## Conclusion

A simple computational approach to local FDR considers a two-components normal mixture model for modeling the observed empirical distribution (*f*) where the null distribution (*f*_0_) is the standard normal and the alternative distribution (*f*_1_) is a normal density with unspecified mean and variance. But the reliability of this approach obviously depends on how well the proposed two-components normal mixture model approximates the real distribution.

Our semi-parametric approach does not assume any constrained alternative distribution and is hence much more flexible. Nonetheless it requires a complete specification of the null distribution, the *a priori *proportion of true null hypotheses (*π*_0_), as well asthe bandwidth (*h*) for which efficient estimation methods have been developed. The performances of the approach compared to existing methods were assessed in a preceding publication [10] which showed its advantages in difficult situations where the distributions *f*_0 _and *f*_1 _are not well separated. We focused here on the implementation of the approach, and on two interesting extensions such as the possibility to use prior information in the estimation procedure (semi-supervised) and the ability to handle truncated distribution such as those generated by Monte-Carlo estimation of *p*-values. Our simulation showed that these informations can significantly improve the quality of estimates. As an illustration, we analyzed three high-throughput biological dataset concerning genes expressions, DNA sequence patterns, and genome-wide association studies. The corresponding R package available at  is fast, thanks to fast Fourier transforms, straightforward to use and propose customizable options to advanced users.

Finally, most of the local FDR estimation procedures derived from the Benjamini and Hochberg framework, including our approach, assume that *p*-values testing true null hypotheses are independent observations. If it may well be the case for patterns, in practice this assumption does not hold for all the genes or SNPs. A proposed solution is to cluster highly correlated genes (or SNPs) together, and to represent a cluster by a single gene or a linear combination of the associated genes [8]. Theses approaches also generally assume that *p*-values testing true null hypotheses are continuous and uniform over [0,1]. These issues are likely to be alive fields of research in the near future.

## Methods

### Probit or logarithm transformations

While it is obviously possible to work directly with a sample of *p*-values (in this case, *f*_0 _is simply the uniform density over [0, 1]) this option is seldom used in practice. This comes from the fact that most **H**_1 _*p*-values are concentrated near 0 while **H**_0 _ones are uniformly distributed between 0 and 1. Working with the rough *p*-values will hence favor estimation of *f*_0 _over *f*_1 _which is precisely our opposite goal. In order to overcome this problem it is then classical to introduce a transformation that will allow us to "zoom" on the interesting part of the distribution. We propose here to consider two such transformations:

#### Probit transformation

*X *= probit(*P*) = Φ^-1^(*P*)

where *P *is a *p*-value and F is the cumulative distribution function of the normal distribution. If *P *~U ([0, 1]), *X *follows a normal distribution and

$$f_{0}(x)=\varphi (x)=\frac{1}{\sqrt{2\pi }}e^{-\frac{x^{2}}{2}}$$

#### Logarithmic transformation

*X *= log_10_(*P*)

If *P *~U ([0, 1]) the - log(10) × *X *has an exponential distribution and we easily get that

$$f_{0}(x)=\{\begin{array}{ll} \log(10)\times e^{-\log(10)x} & \text{if }x⩽0\\ 0 & \text{else} \end{array}$$

Two assets of this transformation are to give more weight to small *p*-values and to be easier to interpret than the probit transformation (*X *= -2 correspond to *P *= 10^-2^, *X *= -5 to *P *= 10^-5^).

### Estimation of *π*_0_

For all 0 ≤ *λ ≤* 1 we have

$$q=\mathbb{P}(X⩾T(\lambda ))=\pi_{0}\underset{q_{0}}{\underset{︸}{\int_{T(\lambda )}^{+\infty }f_{0}(x)dx}}+\pi_{1}\underset{q_{1}}{\underset{︸}{\int_{T(\lambda )}^{+\infty }f_{1}(x)dx}}$$

where *T *is either the probit or the log_10 _function. We hence get

$$\pi_{0}=\frac{q-q_{1}}{q_{0}-q_{1}}$$

We have *q*_0 _= 1 - *λ *but *q*_1 _is unknown. We notice that the higher *λ*, the closer to 0 *q*_1 _will be. As we can estimate *q *from a sample *X*_1_,..., *X*_*n *_by

$$\hat{q}=\frac{1}{n}\sum_{i=1}^{n}I_{X_{i}⩾\lambda }$$

we obtain the following (conservative) estimator:

$$\hat{\pi_{0}}=\frac{\hat{q}}{1-\lambda }$$

which satisfies *π*_0 _= $\hat{\pi_{0}}$ + *O*(*q*_1_).

It is therefore necessary to find a tradeoff between the magnitude of the error *O*(*q*_1_) (lowest for *λ *= 1.0) and the quality of the estimation $\hat{q}$ (best for *λ *= 0.0).

Storey [17] first proposed to use *λ *= 0.5 which appears to be a good choice in most cases.

### Determination of the bandwidth

About the choice of the bandwidth, our first approach consists in selecting *h *as if we were applying a kernel estimation over the whole sample.

For that matter, the literature proposes many methods already implemented in R: biased and unbiased cross-validation estimations (bcv and ucv), method using estimation of derivatives from [18] (sj-ste for solve-the-equation and st-dpi for direct-plugin) and, in two simple heuristics in the special case of Gaussian kernels: nrd0 from [19] (page 48) and nrd from [20].

### Estimation of *f*_1_: Convolution and Fast Fourier Transforms

If we have an observed sample *x*_1_,..., *x*_*n *_with weights *τ*_1_,..., *τ*_*n *_we get for all *x *∈ ℝ

$$\hat{f_{1}}(x)=\frac{1}{h}\sum_{i=1}^{n}\frac{\tau_{i}}{\tau }K\left(\frac{x-x_{i}}{h}\right)$$

where *τ *= ∑_*i *_*τ*_*i *_and *K *states for the kernel function.

The naive computation of all $\hat{f_{1}}$ (*x*_*i*_) requires a quadratic complexity. Fortunately, [21] introduced an algorithm (later modified by [22]) based on Fast Fourier Transform (FFT, see [23] chapter 12) allowing to perform the same computation with a far more efficient linear complexity (see [23] chapter 13 for more details on fast discrete convolution through FFT).

### kerfdr and discrete *p*-values

In developing their original FDR-control procedure, Benjamini and Hochberg [2] assumed that *p*-values testing true null hypotheses are independent observations from a continuous uniform distribution over [0,1]. A large family of succeeding methods requires the same conditions, to which kerfdr belongs. However, how the performance of these methods are affected when the assumption of continuity or uniformity are violated has not been often considered, contrary to the assumption of independence (see [24] and [25] for instance). Discrete *p*-values that become more frequently encountered in practice as categorical genomic data, such as Single-Nucleotide-Polymorphisms, Comparative-Genomic-Hybridation and Copy-Number-Variation become more widely available, clearly violate the assumption of uniformity and introduces instability into FDR-like and local FDR estimates.

In kerfdr, *π*_0 _and the shape of *f*_0 _are parameters of the method. Since with discrete *p*-values, correct estimators of *π*_0 _and *f*_0 _are tricky to obtain with classical methods included in the package, it is still feasible to use methods more adapted to each situation, such as those proposed by [26-29], in order to pre-compute *π*_0 _and/or *f*_0 _before running kerfdr and to minimize the problems generated by discrete *p*-values. However, how our algorithm behaves exactly in this context has still to be considered along with its extension dependent data.

For instance in Figure 7, the short decrease in local FDR observed for the *p*-values near 1 should be interpreted as a nuisance effect that can happen due to a more severe discreteness of *p*-values near 1 (here computed by Monte-Carlo simulations) and hence should be ignored by the user.

## Availability and requirements

Project name: kerfdr

Project home page: 

Operating system: platform independent

Programming language: R

License: GNU GPL

## Authors' contributions

MG most of the redaction, management of the R package (CRAN), application to genome-wide association data. AC estimation of *π*_0_, redaction. SR simulation study, application to gene expression data. GN the kerfdr algorithm (based on FFT convolution), extension of the mixture model to truncated data, application of kerfdr to patterns in DNA sequences.
